# Subtle changes of the crystalline lens after cycloplegia: a retrospective study

**DOI:** 10.1186/s12886-021-01884-9

**Published:** 2021-03-06

**Authors:** Cheng Dai, Meng Liu, Xiaodong Lv, Binzhong Li

**Affiliations:** 1 School of Basic Medicine, North Sichuan Medical College, Sichuan Province 637000 Nanchong, China; 2 Department of Ophthalmology, Affiliated Hospital of North Sichuan Medical College, Sichuan Province 637000 Nanchong, China; 3 Department of Clinical Medicine, North Sichuan Medical College, Sichuan Province 637000 Nanchong, China

**Keywords:** Cycloplegia, CASIA2, Lens biometry, Ametropia

## Abstract

**Background:**

The purpose of this study was to evaluate the shape of the crystalline lens in terms of biometry and diopters before and after cycloplegia using the CASIA2 swept-source (SS) optical coherence tomography (OCT) system on the anterior segment.

**Methods:**

This was a retrospective study. Children and adolescents (26 males and 29 females, aged 4–21 years) with simple ametropia were selected for optometry and CASIA2 imaging at 2 separate visits before and after cycloplegia. Diopter values were derived from the spherical power (S) obtained by optometry. Biometric parameters of the crystalline lens, including the anterior chamber depth (ACD), anterior and posterior curvature of the lens (ACL and PCL), lens thickness (LTH), lens decentration (LD), lens tilt (LT), and equivalent diameter of the lens (LED), were measured by the CASIA2 system. The differences in these parameters after compared with before cycloplegia were determined, and their relationships were analyzed.

**Results:**

Fifty-five participants (106 eyes) were initially enrolled. There was a significant difference (*P* < 0.05) in the S (*t*=-7.026, *P < 0.001*), ACD (*t*=-8.796, *P < 0.001*), ACL (*t*=-13.263, *P < 0.001*) and LTH (*t* = 7.363, *P < 0.001*) after compared with before cycloplegia. The change in the PCL (*t* = 1.557, *P* = 0.122), LD (*t* = 0.876, *P* = 0.383), LT (*t* = 0.440, *P* = 0.661) and LED (*t*=-0.351, *P* = 0.726) was not statistically significant (*P* > 0.05). There was a significant (*P* < 0.05) correlation of the change in the S with that in the ACL (*r* = 0.466, *P < 0.001*), LTH (*r*=-0.592, *P < 0.001*), and LED (*r* = 0.223, *P =* 0.021) but not the PCL (*r* = 0.19, *P =* 0.051), LD (*r*=-0.048, *P =* 0.0628) or LT (*r*=-0.022, *P =* 0.822). Furthermore, the change in the ACD was closely related to the change in crystalline morphology. However, in children and adolescents, we found that the change in crystalline morphology was unrelated to age.

**Conclusions:**

Changes in lens morphology after compared with before cycloplegia are mainly related to the ACL and LTH, but there is no difference in the PCL, LD, LT, or LED. In the adolescent population, change in the S is related to change in the ACL, LED and LTH. However, age is unrelated to the shape and tendency of the crystalline lens. Further research is required to determine whether the same conclusion applies to different age groups and different refractive states (myopia, hyperopia, emmetropia) .

## Background

Since children and adolescents have a strong adjustment ability, cycloplegia is necessary to eliminate the interference of accommodation and obtain accurate diopter measurements [1]. Therefore, optometry after cycloplegia has always been the focus of refractive epidemiological research [2]. How does the crystalline morphology change after cycloplegia is induced? Is this change related to age or diopter measurements? Due to the lack of equipment for directly measuring parameters of crystalline morphology, there have been few related studies. The CASIA2 system is a new type of anterior-segment scanner that can produce reliable in vivo lens measurements regardless of the accommodation stress; the measurement of anterior-segment parameters by this system is not subject to pupil dilation, and the measurements show good repeatability and reproducibility [3–5]. Using the CASIA2 system, applying the same pattern of examination before and after cycloplegia can help us to better observe and understand the changes in the lens and provide a reference for research on myopia and accommodation.

## Methods

### Subjects

This was a retrospective analysis of 55 patients (106 eyes), including 26 males and 29 females, with an average age of 9 ± 3.25 years; 43 (82 eyes) were aged 4–11 years, 9 (18 eyes) were aged 12–18 years, and 3 (6 eyes) were aged 19–21 years. All patients were admitted to the Department of Ophthalmology, Affiliated Hospital of North Sichuan Medical College from May to June 2020. All patients had simple ametropia. Patients with organic diseases, such as those affecting the ocular surface and fundus, and patients who had undergone operations affecting refraction, the ocular surface or the fundus in the past were excluded.

### Methods

Before and after cycloplegia, biometric parameters of the lens were measured by the same experimenter. An ARK-510A autorefractor (NIDEK, Co., Ltd, Gamagori, Japan) and DK-700 optometry system (Topcon, Japan) were used to perform standardized optometry, and the CASIA2 system (Tomey Corp., Nagoya, Japan) was applied to obtain other measurements. The S, ACD, ACL, PCL, LTH, LD, LT and LED were recorded. Cycloplegia was induced with 0.5 % tropicamide eye drops (Boshlen Frida Pharmaceutical Company) applied once every 10 minutes for a total of three times, and relevant examinations after cycloplegia were carried out 30 minutes later. This was a retrospective study. As this extended retrospective study of relevant approved prospective studies [2020ER(A)068] does not harm the interests of the patients, it was exempted by the Ethics Committee of North Sichuan Medical College.

### Statistical analysis

SPSS 25.0 statistical software was used for analysis. All data were first tested by the Kolmogorov-Smirnov (*K*-*S*) goodness-of-fit normality test before comparisons between before and after cycloplegia were made. Data with a normal distribution before and after cycloplegia were compared using a paired sample t test. If the distribution was not normal, a nonparametric test was used for comparison. In the correlation analysis, data with a normal distribution are presented as the mean ± standard deviation (*X* ± *S*), a Pearson correlation analysis was applied, and a scatter plot was used to describe the correlation. If the data did not conform to a normal distribution, the median ± quartile spacing (*M* ± *Q*) was used, and a Spearman correlation analysis was carried out. Multivariate linear regression analysis was performed for parameters with multiple correlations. *P* < 0.05 was considered to indicate statistical significance.

## Results

### Changes in parameters after cycloplegia

**Fig. 1:**
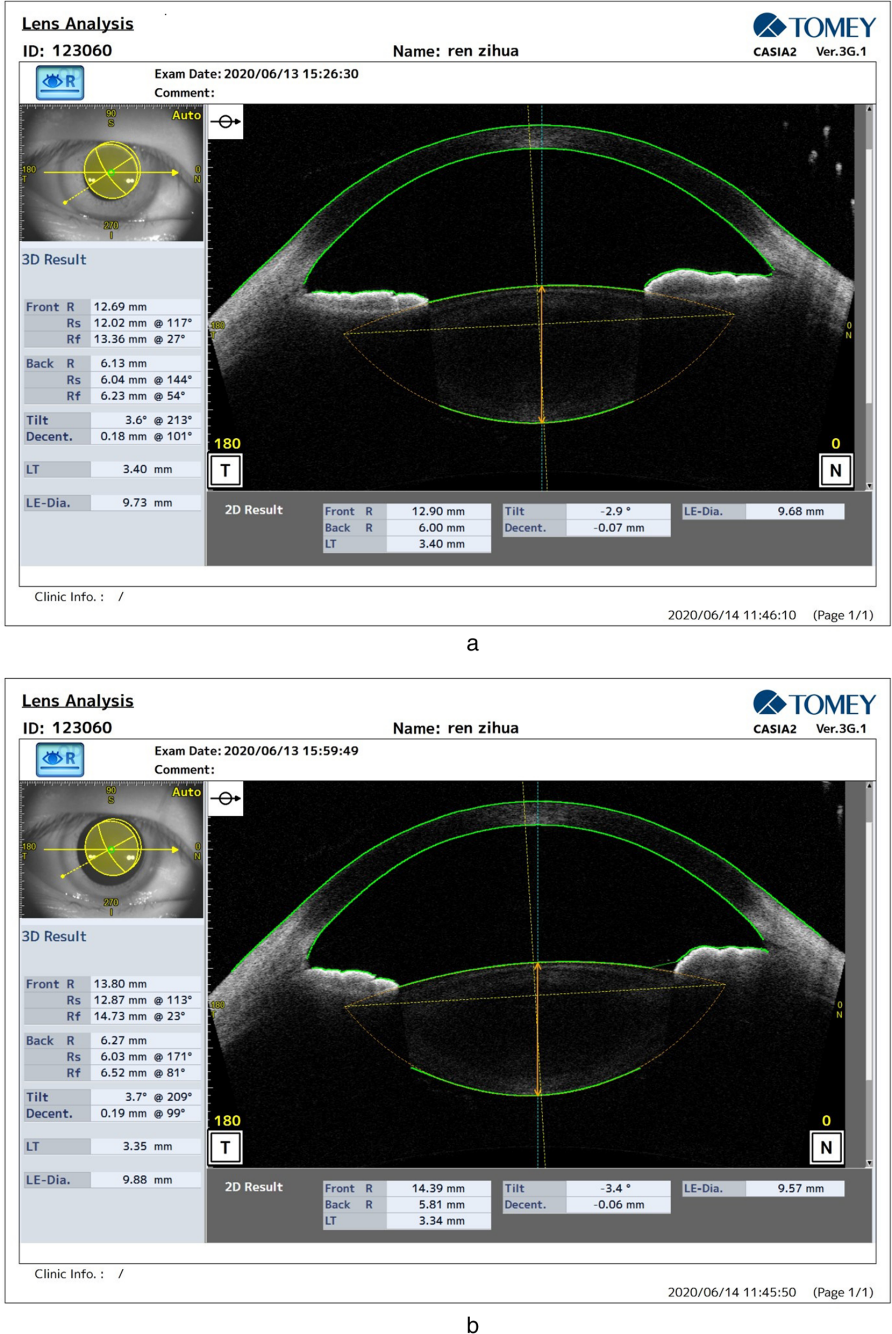
Changes in lens morphology after compared with before cycloplegia **a**: before; **b**: after

Based on data from the CASIA2 system, changes in morphological parameters of the lens after compared with before cycloplegia are shown in Fig. 1a-b. The distribution of biological parameters of the eye before and after cycloplegia are shown in Table 1. There was a significant difference in the S, ACD and ACL and LTH after cycloplegia (*P* < 0.05), while there was no significant difference in the PCL, LT, LD or LED (*P* > 0.05). The PCL, LD and LED showed a normal distribution before and after cycloplegia, while the other parameters showed a skewed distribution, as shown in Table 2.

**Table 1:** The distribution and comparison of all parameters before and after cycloplegia

	Before Cycloplegia		After Cycloplegia		*t/z,P*
	Distribution	Description	Distribution	Description	
S(D)	Skewed	-1.25 ± 2.00	Normal	-0.73 ± 2.13	-7.026,<0.001*
ACD(mm)	Normal	3.20 ± 0.29	Normal	3.26 ± 0.27	-8.796,<0.001*
ACL(mm)	Normal	12.39 ± 1.38	Normal	13.22 ± 1.18	-13.263,<0.001*
PCL(mm)	Normal	5.94 ± 0.46	Normal	5.90 ± 0.46	1.557,0.122
LTH(mm)	Normal	3.47 ± 0.15	Normal	3.44 ± 0.14	7.363,<0.001*
LT(°)	Normal	4.80 ± 1.23	Normal	4.77 ± 1.22	0.440,0.661
LD(mm)	Normal	0.22 ± 0.08	Normal	0.21 ± 0.09	0.876,0.383
LED(mm)	Normal	9.56 ± 0.35	Normal	9.57 ± 0.34	-0.351,0.726

*It indicates that the comparison is statistically significant

**Table 2:** The description of the change of all parameters before and after cycloplegia

	Changes	
	Distribution	Description
S(D)	Skewed	0.25 ± 0.50
ACD(mm)	Skewed	0.045 ± 0.06
ACL(mm)	Skewed	0.69 ± 0.72
PCL(mm)	Normal	-0.04 ± 0.03
LTH(mm)	Skewed	-0.03 ± 0.04
LT(°)	Skewed	-0.007 ± 0.008
LD(mm)	Normal	0.1 ± 0.6
LED(mm)	Normal	0.006 ± 0.018

### Relationship between change in lens biometry and change in S

The change in the S of all 106 eyes was as follows: -0.25 (4 eyes), 0 (33 eyes), 0.25 (32 eyes), 0.50 (15 eyes), 0.75 (11 eyes), 1.00 (5 eyes), 1.25 (1 eye), 1.50 (1 eye), 1.75 (1 eye), 2.00 (1 eye), 2.25 (1 eye), and 3.00 (1 eye). The change in the S was positively correlated with the change in the ACL (*r* = 0.466, *P < 0.001*) and LED (*r* = 0.223, *P* = 0.021). There was a negative correlation with the change in the LTH (*r*=-0.592, *P < 0.001*), but there was no correlation with the change in the PCL (*r* = 0.19, *P* = 0.051), LD (*r*=-0.048, *P* = 0.0628) or LT (*r*=-0.022, *P* = 0.822).

### Relationship between change in lens biometry and change in ACD

The change in the ACD was positively correlated with the change in the ACL (*r* = 0.584, *P < 0.001*) and negatively related to the change in the LTH (*r*=-0.587, *P < 0.001*). However, there was no correlation with the change in the PCL (*r* = 0.035, *P* = 0.725), LD (*r* = 0.022, *P* = 0.821), LT (*r* = 0.018, *P* = 0.858) or LED (*r* = 0.086, *P* = 0.382).

### Relationship between change in lens biometry and age

Age was not related to the change in the ACD (*r* = 0.012, *P* = 0.901), ACL (*r* = 0.067, *P* = 0.496), PCL (*r*=-0.052, *P* = 0.595), LTH (*r* = 0.04, *P* = 0.685), LD (*r*=-0.026, *P* = 0.788), LT (*r*=-0.132, *P* = 0.178), or LED (*r*=-0.029, *P* = 0.77).

## Conclusions

There have been few studies focusing on the observation of refractive power in relation to lens morphology. In this paper, the CASIA2 system was used to compare the parameters of refractive power and crystalline morphology after compared with before cycloplegia. It is well known that ciliary muscle contraction and suspension ligament relaxation alter the surface curvature of the lens, thus increasing the fundamental optical power of the lens [6]. However, cycloplegia results in a hyperopic shift and astigmatic axis change, while the astigmatic power is basically unchanged [7]. The changes in diopters in this paper refer specifically spherical equivalents. Regarding these changes after compared with before cycloplegia, 96.2 % (102 eyes) of 106 eyes showed positive changes to different degrees, while the other 3.8 % (4 eyes) showed a change of -0.25 D. Due to the relaxation of accommodation, the diopters change in the direction of correction. The conclusion of this paper is compatible with those of previous studies and with previous theories [8]. It should be mentioned that there were very few cases with a small amount (-0.25 D) of adverse deviation, which may be due to the following: (1) significant changes in high-order aberrations of eyes after pupil dilation will affect the measurement of diopters to varying degrees [9, 10]; (2) adolescents have an extremely strong accommodation capability, so the results of optometry with a small pupil may not be reliable; and (3) there is a possibility of measurement errors due to the degree of patient cooperation.

The change in crystalline morphology after compared with before cycloplegia was related mainly to the ACL and LTH, but there was no obvious difference in the PCL, LD, LT or LED. Schachar et al [11] reported that during the accommodation process, the ACL changed more than the PCL, while the lens itself had an accommodation range of approximately 7.8 D, and there was no movement or deviation during cycloplegia due to the lack of movement of the lens nucleus. Grzybowski et al [6] also found that a micro increase in lens thickness was related to a large change in accommodation amplitude, while the lens position showed no obvious change. Changes in ocular biological parameters after compared with before cycloplegia have always been a hot topic in refractive research. Different mydriasis drops can achieve dissimilar degrees of regulatory relaxation by paralyzing ciliary muscles [12]. Tadahiro et al [13] evaluated the effect of tropicamide/phenylephrine on ciliary muscle paralysis using the CASIA2 system, and the trends of crystalline changes are consistent with those in this article. In the human eye, the changes in crystalline morphology after compared with before cycloplegia are consistent with the changes in crystalline morphology during dynamic accommodation.

The change in the diopters (S) after compared with before cycloplegia was highly related to the change in the ACL, LTH and LED but was not related to the change in the PCL, LD or LT. Under the conditions of a constant axial length and corneal refractive power, change in the refractive power is mainly determined by change in the lens refractive power. After cycloplegia, the ACL of the crystalline lens increases, and the thickness of the lens decreases. According to the lensmaker equation [14], both increasing the radius of curvature and reducing the thickness of the lens reduce the diopters of the crystalline lens, which corresponds with the change in the diopters. Change in the diameter does not affect change in the lens diopters but is related to change in the diopters. This may be because under the conditions of ciliary muscle relaxation, the radius of curvature of the anterior surface of the lens increases, the thickness of the lens decreases, and the increase in diameter results from a process of overall change. Amy et al [15] reported that the radius of curvature of the rear surface of the crystalline lens changes significantly during the accommodation process. However, we found that the change in the PCL was not related to the change in the diopters and that the change in the PCL after compared with before cycloplegia was not statistically significant. There may be two reasons for these findings. One is that the PCL is small, and the change in its value upon relaxation is not obvious. Second, the circular fibers relaxing in cycloplegia mainly act on the anterior surface of the lens and have little influence on the posterior surface. Changes in the diopters of the human eye are also related to the use of cycloplegic drugs, and the most suitable cycloplegic drugs should be comprehensively selected according to the age, refractive state and other basic conditions of the patient [16, 17].

The change in the ACD after compared with before cycloplegia was highly related to the change in the ACL and LTH but was unrelated to the change in the PCL, LD, LT and LED. Chen, Z et al [18] found that the lens became thinner and moved backward after cycloplegia was induced. The increase in the ACD was primarily due to the backward movement of the lens. These results are worthwhile to clarify the effect of lens-related changes during accommodation.

The changes in the various parameters related to the crystalline morphology of the lens were not related to age. Richdale et al.’s [19] quantitative accommodation study showed that the changes in the equatorial diameter of the lens and ciliary muscle thickness per diopter were not related to the age of the subject and that even if the total accommodation amplitude decreased, the degree of ciliary muscle contraction per diopter was not related to age. In addition, it is possible that this study was mainly focused on adolescent myopia patients, which involves certain age limitations. In patients with myopia, accommodation is often delayed and insufficient [20], with no differences in multiple lens parameters after compared with before cycloplegia.

The CASIA2 system can provide preliminary measurements of changes in biological parameters of the crystalline lens for quantitative and objective evaluation [21], which can help us to better understand changes in various biological parameters of the anterior segment, such as biometric parameters of the lens [22]. Patterns of change in the morphology of the crystalline lens are the basis for research on ametropia, visual regulation and other related aspects and can thus provide a reference for further research on myopia and regulatory mechanisms. Based on the comparison and analysis of diopters and crystalline morphology before and after cycloplegia, it was found that in children and adolescents, the changes in the diopters after compared with before cycloplegia were highly correlated with the changes in crystalline morphology and but not age. Additional research is needed to determine whether the same conclusion can be applied to patients of different age groups and refractive states (myopia, hyperopia and emmetropia).

## Data Availability

The datasets used and analyzed during the current study are available from the corresponding author on reasonable request.

## Abbreviations

SS: Swept-source
OCT: Optical coherence tomography
S: Spherical power
ACD: Anterior chamber depth
ACL: Posterior curvature of the lens
PCL: Posterior curvature of the lens
LTH: Lens thickness
LD: Lens decentration
LT: Lens tilt
LED: Equivalent diameter of the lens
